# Systematic Functional Characterization of Cytochrome *P450 2E1* Promoter Variants in the Chinese Han Population

**DOI:** 10.1371/journal.pone.0040883

**Published:** 2012-07-17

**Authors:** Xunyi Huang, Lili Chen, Wenlong Song, Ling Chen, Jiamin Niu, Xia Han, Guoyin Feng, Lin He, Shengying Qin

**Affiliations:** 1 Bio-X Institutes, Key Laboratory for the Genetics of Developmental and Neuropsychiatric Disorders (Ministry of Education), Shanghai Jiao Tong University, Shanghai, China; 2 Shanghai genomePilot Institutes for Genomics and Human Health, Shanghai, China; 3 School of Life Science and Technology, Shanghai Jiao Tong University, Shanghai, China; 4 Laiwu Hospital, Shandong, China; 5 Institutes of Biomedical Sciences, Fudan University, Shanghai, China; Universidad Europea de Madrid, Spain

## Abstract

*CYP2E1* promoter polymorphisms can lead to significant interindividual differences in expression of CYP2E1. Using a database of *CYP2E1* gene polymorphisms established in 2010, our study aimed to functionally characterize the single nucleotide polymorphisms (SNPs) of the promoter region and corresponding haplotypes in the Chinese Han population. Six novel SNPs and seven haplotypes with a frequency equal to or greater than 0.01 were constructed on a luciferase reporter system on the basis of site-directed mutagenesis. Dual luciferase reporter systems were used to analyze regulatory activity. The constructs including single novel SNP mutations exhibited insignificant change in luciferase activity, whereas, the activity produced by Haplo1(GTTGCTATAT), Haplo2 (CTTGCTATAT) and Haplo7 (GAGCTCACAT), containing a −333T>A polymorphism was significantly greater than for the wild type in Hep G2 cells (p<0.05), being 1.5−, 2.0− and 1.4− times greater respectively. These findings suggest the possibility of significant clinical prediction of adverse drug reaction and the facilitation of personalized medicine.

## Introduction

Human cytochrome P450 2E1 (CYP2E1) plays a central role in the biotransformation of a large number of small molecular weight compounds (eg. drugs, toxins and precarcinogenics) [1]. CYP2E1 can produce reactive oxygen species directly through high oxidative activity and through metabolism of xenobiotics [2]. CYP2E1 levels can be regulated by endogenous and exogenous substrates which might be associated with human susceptibility to toxicity and carcinogenicity caused by industrial and environmental chemicals [3], [4], [5].

In general, genetic polymorphisms are a major determinant of the interindividual variation of CYP450, which leads to the absence of gene product, enzymes with increased, reduced or altered activity, or alteration in enzyme regulation [4]. It has been reported that interindividual variability in *CYP2E1* expression is more than 30 times the corresponding difference in hepatic levels [6], [7]. However, the coding region of *CYP2E1* is highly conserved and it is likely that the variability is related to polymorphic sites in the promoter region [8], [9]. Several reports have demonstrated that *CYP2E1* flanking region polymorphisms cause significant regulation differences. Hu *et al*. [10] functionally characterized the 5′-flanking region in both rat and human cytochrome *P450 2E1* genes, and revealed that 5′-deletion of both the human and rat *CYP2E1* sequences in general caused increased gene expression, indicating negative elements in the more distal parts of the 5′-ﬂanking regions of the genes. Nomura *et al.* [11] found transcriptional activity of the −2363 to −1998 short tandem repeat polymorphism (STR) in the 5′-flanking region of *CYP2E1* to be greater than that of the wild type. However, the single nucleotide polymorphism (SNP) is a major variant form, and it is not known whether the SNPs in −2000 to −1 bp can change promoter activity.

To date, however, no studies have explored the impact on the regulation activity generated by SNP. In a previous systematic screening study we established a database of *CYP2E1* allele frequencies for normal Chinese Han subjects [12]. Based on this work, we analyzed existing haplotypes in the Chinese Han population and built luciferase reporter gene constructs using a site-directed mutagenesis method. We determined the functional significance of the *CYP2E1* regulatory regions through measuring the expression of CYP2E1 in human hepatoma cell lines.

## Results

### *CYP2E1* Regulatory Region Polymorphism Analysis

From the 16 SNPs previously identified by our group in the Chinese Han population [12], 6 newly found SNPs including −1778G>A, −1646A>G, −971A>C, −962A>G, −792T>G, −722A>G, were designed to individually determine their respective regulatory activity (Table 1). The possible haplotypes of the 16 SNPs were analyzed by the online version of SHEsis, a *CYP2E1* SNP database constructed in 2010. 7 haplotypes with frequencies equal to or greater than 0.01 were selected to design linear combination of the SNPs for their functional characterization (Table 2). The haplotypes are consist of 10 of the 16 SNPs (−1653G>C, −1563T>A, −1513T>G, −1293G>C, −1053C>T, −1025T>C, −929A>G, −806T>C, −352A>G, −333T>A, detailed in Table 1). The 6 novel SNPs are not included owing to their low frequencies.

**Table 1:** 16 identified SNPs in the *CYP2E1* promoter region and their frequencies in the Chinese Han population.

Position*	Region	Variant	Minorallele	Allele frequencies (%)
−1778G>A	Promoter	Novel	A	0.1
−1653G>C	Promoter	rs3813865	C	19.7
−1646A>G	Promoter	Novel	G	0.5
−1563T>A	Promoter	rs3813866	A	21.8
−1513T>G	Promoter	rs8192766	G	36.9
−1293G>C	Promoter	rs3813867	C	20
−1053C>T	Promoter	rs2031920	T	20.9
−1025T>C	Promoter	rs2031921	C	21.7
−971A>C	Promoter	Novel	C	0.1
−962A>G	Promoter	Novel	G	0.1
−929A>G	Promoter	rs3813870	G	18.9
−806T>C	Promoter	rs2031922	C	21.5
−792T>G	Promoter	Novel	G	0.4
−722A>G	Promoter	Novel	G	0.1
−352A>G	Promoter	rs2070672	G	18.4
−333T>A	Promoter	rs2070673	A	46.7

* The position in the gene is that indicated by the reference sequence of J02843 in the GeneBank.

**Table 2:** Selected haplotypes of the *CYP2E1* regulatory region in the Chinese Han populations.

Name Haplotypes (−1653 to −333)	Frequency (≥0.01)
Haplo1 GTTGCTATAT	0.556
Haplo2 CTTGCTATAT	0.010
Haplo3 CTTGCTGTGA	0.013
Haplo4 CTGGCTGTGA	0.133
Haplo5 GATCTCACAA	0.013
Haplo6 GAGCTCACAA	0.157
Haplo7 GAGCTCACAT	0.016

### CONREAL Analysis

CONREAL, the tool using biologically relevant information, has been used widely to predict transcription factor binding sites [13]. The CONREAL analysis results showed that the 10 SNPs composing the 7 selected haplotypes in the *CYP2E1* promoter region may alter binding efficiency of the transcription factors (Table 3). Among these transcription factors, it’s worth noting that hepatocyte nuclear factor 4 (HNF-4) is a general regulator supporting the expression of major drug-metabolizing CYPs in human hepatocytes [14], while the redox*-*sensitive transcription factor Sp1 may greatly affect expression and regulation of *CYP2E1*.

**Table 3:** Predictive analysis (performed by CONREAL) of transcription factor binding sites affected by potential regulatory SNPs in *CYP2E1*.

SNPs	Transcription factors with binding efficiency changed				
−1653G>C	CDP		CR3+HD		
−1563T>A	v-Myb		Cap	deltaEF1	
−1513T>G	COUP-TF/HNF-4		COUP-TF	Hand1/E47	
−1293G>C	deltaEF1		Cap		
−1053C>T	TATA	SRY	En-1	CdxA	IRF-1
−1025T>C	TATA		FREAC-7	CdxA	
−929A>G	FREAC-3		SPI-1	STATx	HOXA3
−806T>C	CdxA		Pax-4		
−352A>G	Cap		CP2	CCAAT box	
−333T>A	HNF-4		GC box	Sp1	FREAC-3

### Site-directed Mutagenesis and Construction of pGL3-*CYP2E1* Promoter

**Figure 1:**
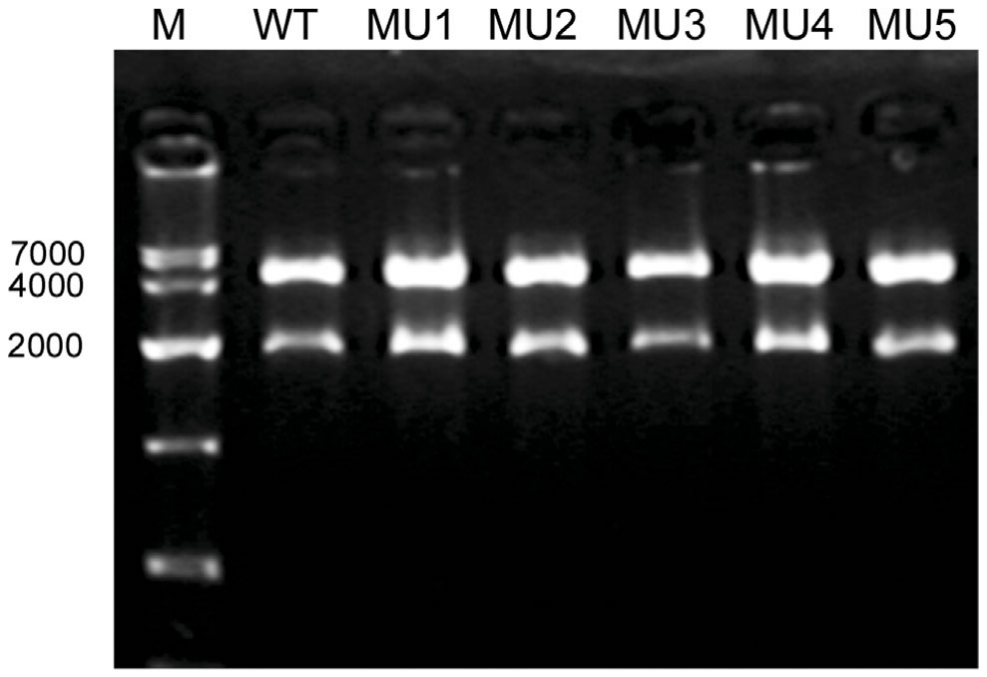
Double restriction enzyme identification of pGL3-*CYP2E1* with *Bgl II* and *Hind III.* WT: wide type; MU1: −1778G>A; MU2: −1646A>G; MU3: −971A>C and −962A>G; MU4: −792T>G; MU5: −722A>G.

1900 bp of the *CYP2E1* 5′-upstream regulatory region had been cloned into PCBG99 vectors at first. Based on the wild type sequence, we designed and synthesized a series of primers for site-directed mutagenesis (Table 4). In this way, 6 novel SNPs were introduced into the PCBG99-*CYP2E1* constructs respectively (with −971A>C and −962A>G in the same construct). For dual-luciferase analysis, we cloned the 6 fragments into pGL3 luciferase reporter vectors. *CYP2E1* promoter constructs were verified by restriction enzyme digestion using *Bgl II* and *Hind III*. The target segment was detected at about 2000 bp (Figure 1). Then the 7 promoter haplotypes were respectively introduced into pGL3-*CYP2E1* constructs by site-directed mutagenesis. Haplotypes in the constructs were verified by sequence analysis.

**Table 4:** Primer sequences for site-directed mutagenesis.

Position	Primer	Sequence
−1778G>A	F	5′−ccagggcacgcaggtcccgctgg−3′
	R	5′−ccagccccacctgcgtgccctgg−3′
−1653G>C	F	5′−tcaccccaccaaagccaacgcttcaatttcagtc−3′
	R	5′−gactgaaattgaagcgttggctttggtggggtga−3′
−1646A>G	F	5′−ccaaagccaaggcttcagtttcagtctgtggggag−3′
	R	5′−ctccccacagactgaaactggaagccttggctttgg−3′
−1563T>A	F	5′−aaacttgtggaccccaaagggtgtctgtccc−3′
	R	5′−gggacagacaccctttggggtccacaagttt−3′
−1513T>G	F	5′−caggacaacagggtgcaggggtctggaca−3′
	R	5′−tgtccagacccctgcaccctgttgtcctg−3′
−1293G>C	F	5′−cttggttcaggagagctgcagtgttaggtgc−3′
	R	5′−gcacctaacactgcagctctcctgaaccaag−3′
−1053C>T	F	5′−ctattatacataaagattcattgttaatataaaagtataaaattgcaacctatgaattaagaactcct−3′
	R	5′−aggagttcttaattcataggttgcaattttatacttttatattaacaatgaatctttatgtataatag−3′
−1025T>C	F	5′−ttgcaacctatgaattaagaactcctatatattgccagttagaagac−3′
	R	5′−gtcttctaactggcaatatataggagttcttaattcataggttgcaa−3′
−971A>C&−962A>C	F	5′−aaaacattctcttcattctaaccccacacacagaaaagctccacaaaatacctatg−3′
	R	5′−cataggtattttgtggagctttttctgtgtgtggggttagaatgaagagaatgtttt−3′
−929A>G	F	5′−acctatggactaccttcgtagaaggtggaagaggg−3′
	R	5′−ccctcttccaccttctacgaaggtagtccataggt−3′
−806T>C	F	5′−agacaagatatctttaaaatcgtcttccaaatttaccctaatgtaaaacaaatcc−3′
	R	5′−ggatttgttttacattagggtaaatttggaagacgattttaaagatatcttgtct−3′
−792T>G	F	5′−tttaaaatggtgttctaaatttaccctaaggtaaaacaaatccaataaaactctaatgt−3′
	R	5′−acattagagttttattggatttgttttaccttagggtaaatttagaacaccattttaaa−3′
−722A>G	F	5′−gaatttaaatttggaataattccaaagaacgatttttcttaatttctacagccagaatata−3′
	R	5′−tatattctggctgtagaaattaagaaaaatcgttctttggaattattccaaatttaaattc−3′
−352A>G	F	5′−agttccccgttgtctagccagtgccaaaggg−3′
	R	5′−ccctttggcactggctagacaacggggaact−3′
−333T>A	F	5′−gccaaagggcaggtcggtacctcaccc−3′
	R	5′−gggtgaggtaccgacctgccctttggc−3′

### Dual-luciferase Analysis

**Figure 2:**
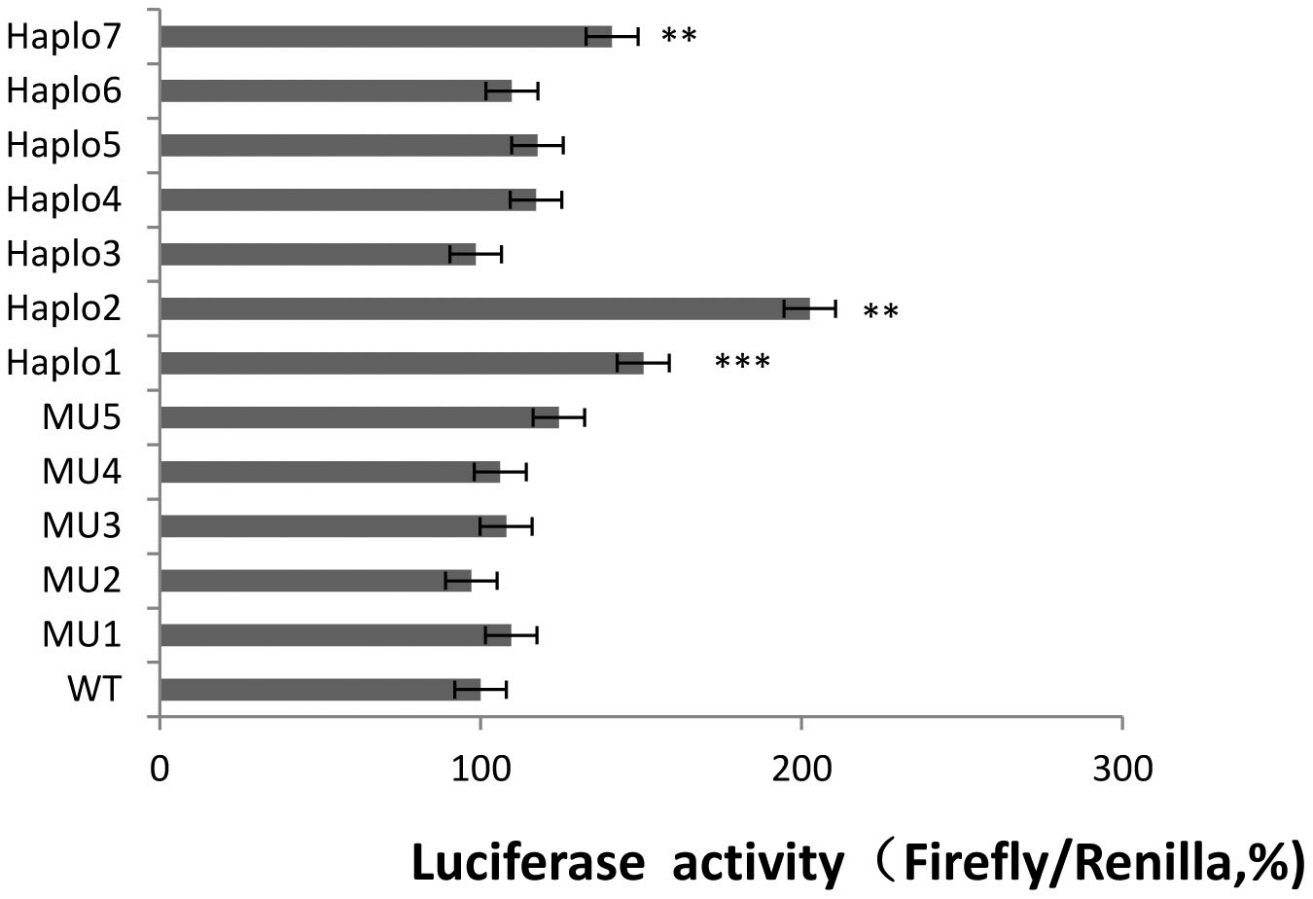
Expression of the human *CYP2E1* reporter constructs in the human hepatoma cell line (Hep G2). The luciferase activities were normalized against the human wild type construct. The results are the average of at least three independent experiments. Comparisons among groups were done with the aid of the ANOVA statistical procedure. **Significantly different from Wild type transfected cells (*P*<0.05), *** Significantly different from Wild type transfected cells (*P*<0.01).

The functional significance of all 6 novel SNPs and 7 haplotypes was determined by the Renilla/Firefly luciferase assay after these constructs were transfected into human hepatoma cell lines. The *Renilla*/Firefly luciferase activity ratio could well indicate the relative activity of *CYP2E1* 5′-upstream regulatory region. 3 haplotype constructs including pGL3-Haplo1 (GTTGCTATAT), pGL3-Haplo2 (CTTGCTATAT) and pGL3-Haplo7 (GAGCTCACAT) exhibited higher luciferase activity as compared to the wild type construct pGL3-WT (*P*<0.05), being 1.5−, 2.0− and 1.4− times greater respectively (Figure 2), whereas the single point mutatations did not appear to change regulatory activity.

## Discussion

CYP2E1 is one of the major phase I drug metabolizing enzymes, although it makes up less than 7% of all the hepatic P450 isoforms [15]. CYP2E1 has a relatively high redox potential as compared to other cytochromes P450 [16] and is easily induced even in the absence of substrates causing the production of reactive oxygen species as it can induce lipid peroxidation, NADPH-dependent peroxidation or other oxidative stress [17]. The enzyme is therefore considered an important source of reactive oxygen species in alcohol-induced liver injury [18]. The phenotypic polymorphism of *CYP2E1* is the likely cause of interindividual drug metabolism differences, xenobiotics-induced liver injury discrepancy, or even severe adverse drug reaction.

Haplotypes exhibiting greater regulatory activity will lead to *CYP2E1* expression diversity, and therefore individuals carrying the corresponding SNPs might have a faster CYP2E1 drug metabolism and a higher risk of xenobiotics-induced liver injury as compared to wild type carriers. The results of this study indicated that the constructs including Haplo1 (GTTGCTATAT), Haplo2 (CTTGCTATAT), Haplo7 (GAGCTCACAT) exhibits up regulation of luciferase activity compared with the wild type construct (Figure 2). Haplo1 (GTTGCTATAT) is a major haplotype present in the Chinese Han population, whereas Haplo2 (CTTGCTATAT) and Haplo7 (GAGCTCACAT) are rare (Table 2). So this finding may contribute to the development of personalized medicine and drug design, as individual differences in CYP2E1 metabolism can be regarded as a predictive factor in prescribing drug dosage to achieve effective therapy.

Transcriptional control of adult rat hepatic *CYP2E1* is exerted by HNF-1 [19], [20], and several other factors are known to govern *CYP2E1* expression in hepatic or extrahepatic tissues. Peng *et al.* [21] reported that Sp1 binding to a 32-base-pair element is a transcription activator for the induction of *CYP2E1* by IL-1 alpha in Hep G2 cells. It is notable that the position −333 binds to Sp1 transcriptional factor. Refer to Table 2, all of the 3 haplotypes contain an A>T mutation at −333. So we infer that Sp1 may play an important role in increasing *CYP2E1* promoter activity. However, the binding efficiency change caused by a single nucleotide mutation might not be enough to alter overall promoter activity. The combination effect of several mutations might be more influential. Additional studies are needed in this area.

In conclusion, this is the first study to carry out a systematically functional characterization of the promoter region of *CYP2E1* variants in the Chinese Han population. This could bring the clinical relevance for the design of personalized medicine regimes and the reduction of adverse drug reactions.

## Materials and Methods

### *CYP2E1* Regulatory Region Polymorphisms Analysis

In a previous systematic screening study by our group, 16 different *CYP2E1* regulatory region polymorphisms, including 6 novel variants in promoter regions, were identified [12]. We analyzed the possible haplotypes of the 16 SNPs by the online version of SHEsis, our 2010 systematically constructed *CYP2E1* SNP database of the Chinese Han population (http://analysis2.bio-x.cn/myAnalysis.php, by Shi YY and He L). 10 of the 16 SNPs comprise the 7 selected SNP haplotypes according to their relatively high frequencies in the Chinese Han populations (Table 1). The 6 novel SNPs with low frequencies were determined individually for regulatory activity.

### Analysis of Functional Sequences in the Promoter Region

The transcription factor binding analysis of the polymorphisms in the promoter region was performed by the web-based CONREAL server: Conserved Regulatory Element anchored Alignment (http://conreal.niob.knaw.nl/).

### Construction of pGL3-*CYP2E1* Promoter

1900 bp *CYP2E1* 5′-upstream regulatory region of *CYP2E1* was amplified from human genomic DNA by nested PCR and cloned to PCBG99 vectors. To detect the regulatory activity, we constructed pGL3-*CYP2E1*, which contains a luciferase reporter gene. The pGL3-Basic and control plasmids were purchased from Promega.

### Site-directed Mutagenesis

To construct the haplotype in vitro, the target SNP sites were introduced by site-directed mutagenesis, the primer for which was designed using a QuickChange Primer Design tool. PCR was carried out in the 10xpyrobest buffer consisting of 1 mM of dNTP mix, 15 pM of each primer, 1.5 U pyrobest polymorphrase (Takara) in a volume of 15 µl with initial denaturation for 1 min at 95°C, followed by 18 cycles of denaturation at 95°C for 50 sec, annealing at 60°C for 50 sec and extension at 68°C for 7.5 min. A final extension for 7 min at 68°C was performed after the last cycle.

### Cell Culture and Transfection

The human hepatoma cell (Hep G2) provided by ATCC, was cultured as a monolayer in a Dulbecco minimal essential medium containing 15% fetal bovin serum and 100 µg/ml each of penicillin and streptomycin in a humidified atmosphere containing 5% CO_2_. The cells were grown in 25 cm^2^ ﬂasks to 90−100% conﬂuence and then divided and placed in 2.0 cm^2^ culture dishes (costar) for experimental usage. These cells were allowed to grow into conﬂuence and then cultured for an additional 2–3 weeks before the experiments were processed. 0.5 µg of each *CYP2E1* construct containing fireﬂy luciferase reporter gene was transfected into the Hep G2 cells using Transfectin (Bio-rad). The pRL-SV40 plasmid (Promega) (1/10 pGL3-*CYP2E1* construct) containing the Renilla luciferase reporter gene was co-transfected with the pGL3-*CYP2E1* constructs to provide an internal control of the transfection efficiency. 8 replicates per sample were performed.

### Dual-luciferase Reporter Assay

The cells were incubated with the lipid-DNA complexes for 24−72 h. The culture medium was removed and washed with 200 µl PBS per each well, and the fireﬂy and Renilla luciferase activities were then detected using the Dual Luciferase reporter assay system kit (Glomax multi-detection system, Promega).

### Statistical Analysis

The ratio between the fireﬂy luciferase activity and the Renilla luciferase activity was used to analyze relative activity. All statistical analyses were performed using a two-tailed independent sample t-test. All results are expressed as mean ± SD and compared with controls. A p value of <0.05 was considered statistically significant.
